# Advances in the development of a dissolution method for the attribution of iridium source materials

**DOI:** 10.1007/s10967-016-5151-4

**Published:** 2017-01-21

**Authors:** A. P. J. Hodgson, K. E. Jarvis, R. W. Grimes, O. J. Marsden

**Affiliations:** 1 AWE plc, Aldermaston, Reading, Berkshire RG7 4PR UK; 2 Department of Materials, Royal School of Mines, Imperial College London, Exhibition Road, London, SW7 2AZ UK; 3 Centre for Environmental Policy, Faculty of Natural Sciences, Imperial College London, South Kensington Campus, London, SW7 1NA UK

**Keywords:** Iridium targets, Dissolution, Pressure vessels, Speciation, Nuclear forensics

## Abstract

To assist in nuclear forensic investigations, new techniques are required to evaluate radioactive materials that may be discovered outside of regulatory control. Using a recently developed pressure digestion method for iridium powder, assessments have been made of this techniques suitability for undertaking iridium target material evaluations. In addition to determining the reaction conditions necessary for total dissolution, these investigations have provided an insight into the elemental impurities that are present within unirradiated iridium targets that are used in QSA Global radiography sources, and established the speciation of the iridium solutions that are formed during this process.

## Introduction

A number of well documented events where radiological sources have been discovered outside of the regulatory system have helped to substantiate the belief that radioactive materials are open to being exploited for malicious intent [1–5]. Being able to distinguish between different material production routes and locations, through the use of nuclear forensic signatures, could therefore be essential in attributing any such material back to its source of origin [6].

All of the radioisotopes that authorities deem as being of greatest security concern are generated in nuclear reactors via either neutron activation of elemental targets, or by fission/activation reactions in actinide materials. The radioisotopes that are produced directly from the irradiation of stable elemental targets [cobalt-60 (^60^Co) and iridium-192 (^192^Ir)] are of particular interest, because they are not available carrier free (Table 1). Unlike radioisotopes generated from fission/actinide activation reactions, these materials undergo no post production processing. Other unintended nuclides generated during the course of the irradiation process will therefore remain embedded in the final source material produced. As such, these nuclides could be useful in helping authorities establish the processing history of these materials if they are discovered outside of regulatory control.

**Table 1:** ^60^Co and ^192^Ir nuclear data

Radioisotope	Half life	Typical specific activity (GBq/g)	Radiation emissions
^60^Co	5.27 years	41,800	High energy gamma (γ) Low energy beta (β)
^192^Ir	73.83 days	37,000	High energy γ High energy β

In order to identify which nuclides could potentially be used as nuclear forensic signatures, computational studies are being undertaken by the authors to understand how the impurity nuclides in these two materials alter with reactor environment and the irradiation process used. To substantiate any conclusions that are drawn from these investigations, the validity of the models must be confirmed through the examination of materials of known production history. For this to occur, chemical dissolution, separation and purification methods must be used to separate the impurity nuclides from the material’s main radioactive constituent. In the case of iridium this can prove difficult to achieve.

To ensure that nuclides are not masked by background impurities, iridium dissolution techniques using only high purity reagents are necessary. Although methods are available, these are all focused towards geological sample types, which only contain trace levels of iridium [7–9]. The authors have therefore been developing a new method for digesting samples where iridium is the main constituent. Using pressure digestion, and hydrogen peroxide (H_2_O_2_) as an oxidising agent, this method has been shown to be capable of dissolving iridium powder [10].

The reaction mechanism between H_2_O_2_ and hydrochloric acid (HCl) is best described by the equations:

1 $${\text{H}}_{ 2} {\text{O}}_{ 2} + {\text{ Cl}}_{ 2} \leftrightarrow {\text{H}}^{ + } + {\text{ Cl}}^{ - } + {\text{ HClO}}_{ 2}$$

2 $${\text{HClO}}_{ 2} \to {\text{O}}_{ 2} + {\text{ H}}^{ + } + {\text{ Cl}}^{ - } .$$

At the high acid concentrations experienced during pressure digestion, the formation rate of oxygen (O_2_) and chloride ions (Cl^−^) is only limited by the decomposition rate of chlorous acid (HClO_2_) [11]. As the chemistry of iridium–chloro complexes in solution are still not that well defined, the impact that sample oxidation has on the formation of iridium(III) and iridium(IV) species is unclear [12]. Determining the iridium species formed during the dissolution process is therefore key, because this will dictate the separation chemistry required to isolate and purify the impurity nuclides of interest. Further studies have consequently now been performed to confirm the speciation of the sample solutions generated, and to assess the techniques suitability for source material evaluations. Details of these investigations are provided here, together with the results of initial assessments that have been made into iridium target material impurities.

## Experimental

### Apparatus

#### Digestion

Digestec DAB-2 pressure vessels (BERGHOF, Products + Instruments GmbH) were used to perform iridium target material digestions. Samples, together with the reagents, were placed in 50 mL Teflon^®^ TFM PTFE liners before being sealed in the stainless steel pressure vessels using bayonet style closure devices. External heating was provided by the DAH-406 heating block (BERGHOF, Products + Instruments GmbH), and the temperature of the system was monitored and regulated by the BTC-3000 temperature regulator (BERGHOF, Products + Instruments GmbH).

Non-destructive elemental analysis of the target surfaces were made prior to dissolution using an ARTAX micro-focus X-ray Fluorescence (XRF) spectrometer (Bruker Corporation).

#### Elemental determinations

The concentration of iridium and a number of potential elemental impurities [copper (Cu), gold (Au), osmium (Os), palladium (Pd), platinum (Pt), rhenium (Re), rhodium (Rh), ruthenium (Ru), silver (Ag) and tungsten (W)] were determined using an X Series II inductively coupled plasma-mass spectrometer (ICP-MS) (Thermofisher Scientific). To offset iridium’s significant memory effect on the instrument, an extended washout sequence involving the use of 10% HCl for 120 s, followed by water and 5% HCl for 30 and 25 s, respectively, was required.

A solution containing Indium-115 and Thallium-204, prepared from their respective 1000 μg mL^−1^ single-element standard solutions (VHG Labs, LGC Standards), was used to correct for instrumental drift and sensitivity. For each element analysed, external calibration was performed using a series of matrix-matched standards that covered the expected concentration range. In general this was 0.1–20 and 1–100 ng mL^−1^ for the impurities and iridium, respectively. In all instances, except osmium and platinum where the isotopes measured were limited due to the formation of iridium hydrides, elemental determinations were made based on the natural composition of the element.

#### Speciation

Variations in iridium speciation were assessed using an 8453 diode array ultraviolet (UV)–visible (Vis) spectrophotometer (Agilent Technologies) and an iHR 320 Raman system (Horiba Jobin Jvon Ltd).

To reduce the chances of fluorescence during Raman evaluations, sample solutions and standards were dried under vacuum. Analysis was then performed under ×50 magnification using a 523 nm green laser, which when combined with the optimisation of the system power (4.5–9.0 mW) and spectral collection time (30–200 s) ensured the production of detailed spectra with the highest possible signal to noise ratio. To avoid saturating the photodiode detector of the UV–Vis spectrophotometer, sample solutions were arbitrarily diluted using concentrated HCl (c.HCl) prior to analysis. The wavelength range (190–1100 nm) and resolution (better than 2 nm) of the system meant the spectrophotometer was then able to provide rapid sample screening together with excellent sensitivity and near absolute wavelength reproducibility.

### Procedure

Samples were weighed into 50 mL Teflon^®^ TFM PTFE liners, together with 5 mL c.HCl and either 100 or 150 μL of H_2_O_2_. The liners were then placed in the pressure vessels and sealed before being heated to either 210 or 230 °C for a predefined time period. A process blank, consisting of only c.HCl and H_2_O_2_ was run with each batch. Once the vessels had cooled to room temperature, they were disassembled and the solution separated from any residual material by centrifugation. In those instances where there was minimal dissolution, sample residues were crushed using a boron carbide pestle and mortar prior to further digestion attempts.

For each solution generated by a sample, iridium concentrations (ng mL^−1^) were determined by ICP-MS using serially diluted sub-samples. These results were then used to calculate the amount of iridium that had dissolved, by comparing against the amount of material originally added. The Raman and UV–Vis spectra generated by each solution also enabled qualitative speciation assessments to be made against an iridium reference standard.

Once fully dissolved, and the iridium concentrations known, all the aliquots generated by each target material were bulked together. An ICP-MS assessment was then performed to evaluate the elemental concentration (ng mL^−1^) of any impurities present, and then converted back to per gram of original material (μg g^−1^).

### Reagents

Target material dissolutions were performed on stable iridium pellets (1.4 × 1.4 mm) and discs (2.7 × 0.125 mm) that are manufactured by QSA Global for use in radiography sources following irradiation. The solutions and standard used in sample speciation studies were prepared using 99.9% pure trace metal basis iridium powder (Acros Organics) and hydrogen hexachloroiridate(IV) hexahydrate powder (Fisher Scientific), respectively. All other reagents used in these investigations [c.HCl (32–35%) and H_2_O_2_ (30–32%)] were Optima™ grade to reduce the potential for sample contamination. Dilutions of these stock solutions, where required, were prepared using type I water (18.2 MΩ-cm) generated by a Purelab Option-Q instrument (ELGA Labwater).

### Uncertainty

All data analysis was performed on multiple sample measurements (*n* = 10). Uncertainties at the 1*σ* confidence level were generated for each analysis using a combination of the percent relative standard deviation (%rsd) on the measured values and the ICP-MS instrumental precision (as defined by quality controls run alongside the experimental samples).

## Results and discussion

### Sample speciation

**Fig. 1:**
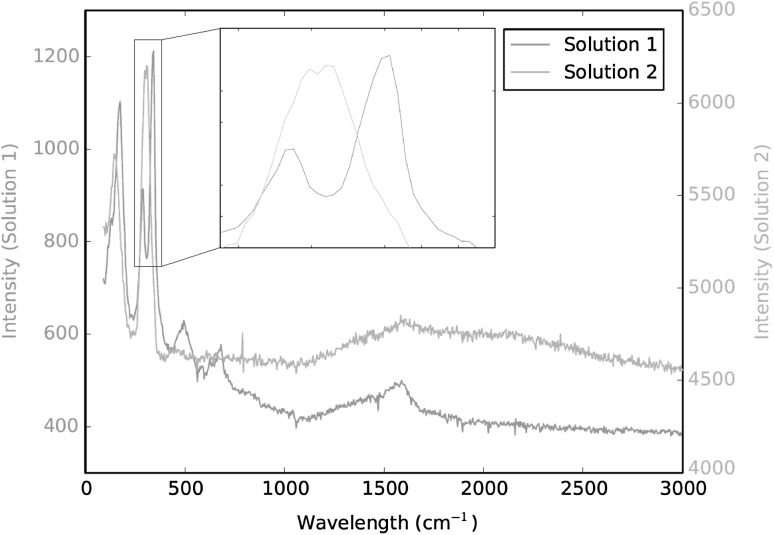
Raman spectra of the solutions produced from fully dissolved (Solution 1) and partially digested (Solution 2) iridium powder under the same conditions

Determining the iridium species that are formed in solution following pressure digestion is essential, as it dictates the separation chemistry that will be required to isolate the impurity nuclides of interest from the main source material. Previous studies using iridium powder have indicated that the species formed is highly dependent on the level of dissolution occurring; with reddish/brown and orangey/yellow coloured solutions being formed during full and partial sample dissolution, respectively [10]. Raman evaluations showed these solutions to have strong similarities, and it was suggested that when full dissolution did not occur an intermediary species was being formed instead of hexachloroiridate(IV). More detailed Raman analyses have reaffirmed this conclusion, as the spectra from fully dissolved sample solutions were seen to exhibit the characteristic 323 cm^−1^ peak of partially digested sample solutions in addition to their own main peak at 346 cm^−1^ (Fig. 1). When an extra 100 μL of H_2_O_2_ was added to the partially dissolved sample solutions, they were all seen to readily convert to the same colorimetric state as the fully dissolved species without the need for any further heating. This colour change therefore suggests the presence of lower oxidation state complexes of iridium in the partially dissolved sample solutions, such as hexachloroiridate(III), which are then being rapidly oxidised to iridium(IV) [13, 14].

**Fig. 2:**
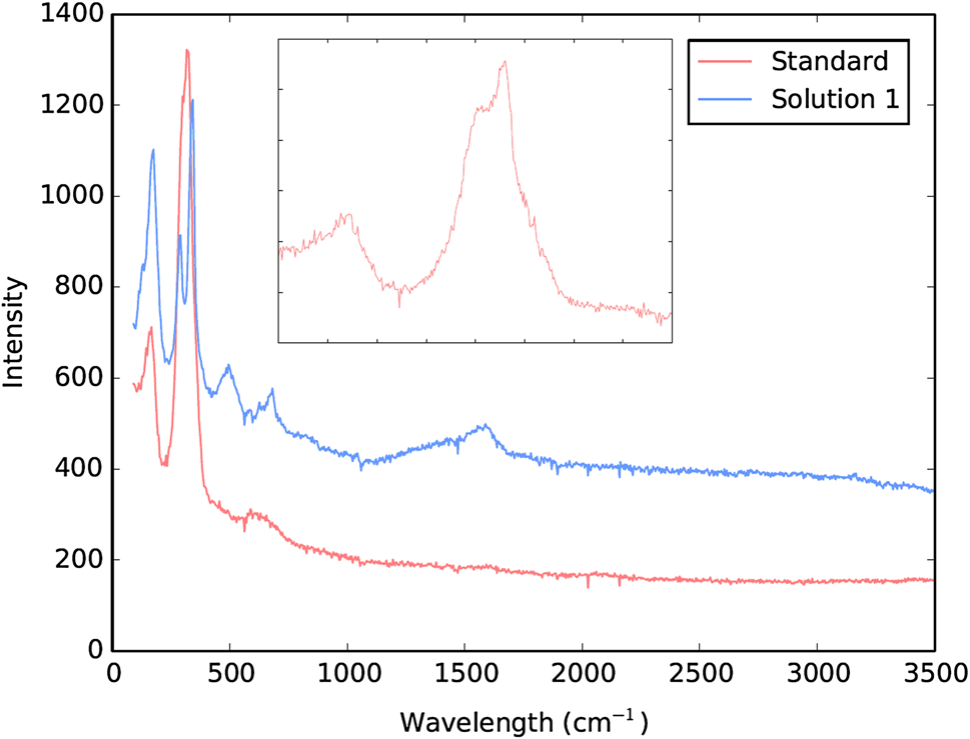
Comparison of hexachloroiridate(IV) reference material and fully dissolved sample solution (solution 1) Raman spectra when analysed under the same conditions; together with the reference standard under full power (*inset*)

Raman spectra of a hexachloroiridate(IV) reference standard have also been shown to correlate well with the spectra generated by the fully dissolved sample solutions (Fig. 2). The broad peak of the reference standard fully incorporates the sample solution’s two main peaks at 323 and 346 cm^−1^ when analysed under the same conditions. It is even possible to begin resolving this broad peak into its two constituent parts by increasing the instrumental power up to 9.0 mW.

**Fig. 3:**
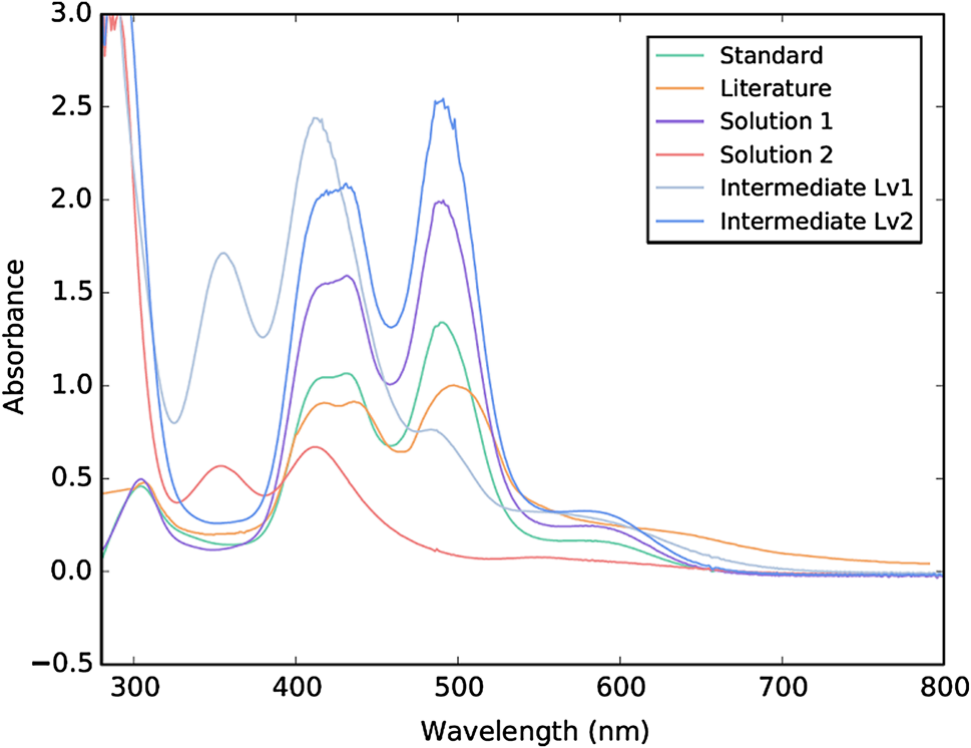
Qualitative assessment of absorption spectra generated by various iridium solutions relative to hexachloroiridate(IV) literature values

Unfortunately, there is currently no Raman data for hexachloroiridate(III) and (IV) available within the literature for comparison. In order to confirm hexachloroiridate production, and to examine the plausibility behind intermediary formation, assessments of the species’ principle absorption bands between 300 and 600 nm were made using UV–Vis spectroscopy [14–17]. These investigations were not only able to confirm the hypotheses, but also highlighted the disparity there can be in iridium speciation between sample solutions prior to total dissolution (Fig. 3). The absorption peaks for hexachloroiridate(IV) from the literature at 304, 418, 435 and 488 nm for instance, are clearly observable in both the reference standard and the fully dissolved solution 1. The absorption peaks for solution 2 at 355 and 415 nm are characteristic of hexachloroiridate(III). Other partially dissolved sample solutions though, such as those indicated by the intermediate level 1 and 2 spectra in Fig. 3, appear to contain hexachloroiridate in a mixture of both the +3 and +4 oxidation states. This suggests that the amount of hexachoroiridate(IV) present is dependent on the extent of dissolution, as this is directly related to the efficiency of sample oxidation.

### Target dissolution

In addition to speciation studies, preliminary evaluations were made of the suitability of the method for digesting actual source materials. Compared to iridium powder, source materials have much smaller surface area to volume ratios. The effect that this has on the oxidising efficiency of H_2_O_2_, and as a consequence the quantity of iridium that is dissolved, requires further investigation.

Initial dissolution runs of QSA Global iridium pellets and disks using the conditions developed for iridium powder (100 μL H_2_O_2_ and a reaction temperature and time of 210 °C and 12 h, respectively) showed minimal dissolution (Tables 2, 3). A series of tests varying either the volume of oxidising agent, or the reaction time or temperature, also showed limited, if any, improvement in sample digestion. The pellets and disks both showed higher levels of dissolution at 230 °C. The dissolution conditions for target materials was therefore modified to reflect this. The volume of oxidising agent was also increased up to 150 μL, because for both target types this resulted in more consistent digestion between dissolution runs when performed over a 12 h time period.

**Table 2:** Iridium pellet recoveries based on the digestion conditions used

Volume of H_2_O_2_ (μL)	Time (h)	Iridium dissolved (%)	
		210 °C	230 °C
100	12	0.35 ± 0.02	0.65 ± 0.05
	24	0.40 ± 0.01	0.70 ± 0.10
150	12	0.25 ± 0.02	0.75 ± 0.05
	24	0.20 ± 0.08	0.80 ± 0.05

**Table 3:** Iridium disk recoveries based on the digestion conditions used

Volume of H_2_O_2_ (μL)	Time (h)	Iridium dissolved (%)	
		210 °C	230 °C
100	12	2.00 ± 0.08	3.70 ± 0.20
	24	2.50 ± 0.25	6.90 ± 2.60
150	12	1.70 ± 0.40	5.45 ± 0.25
	24	0.90 ± 0.40	3.80 ± 0.85

**Fig. 4:**
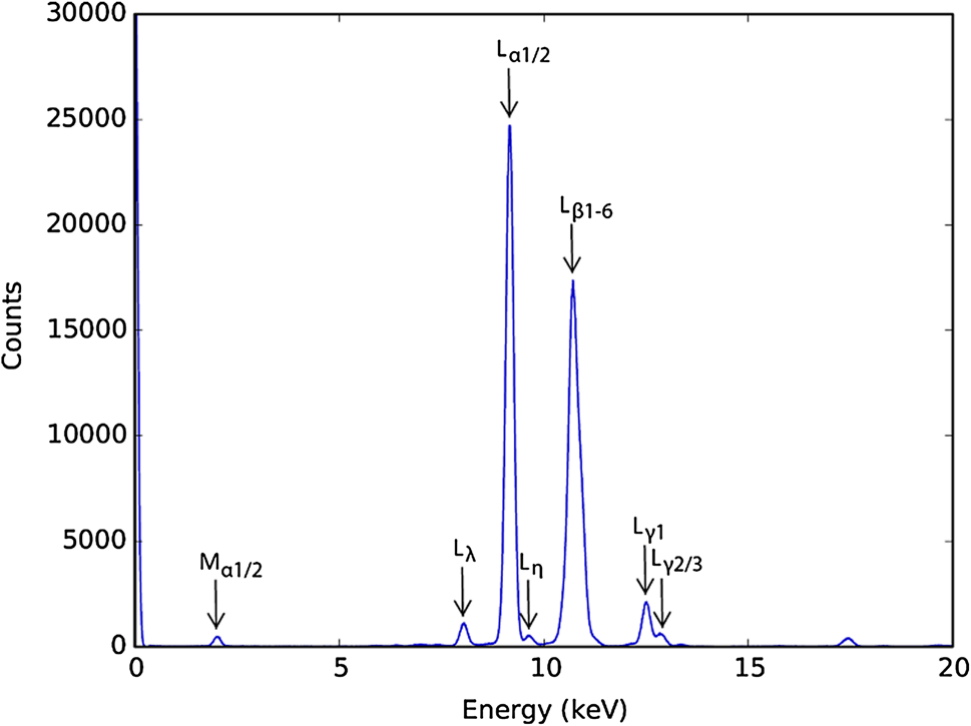
Typical spectra and corresponding *L* and *M* lines generated by iridium pellets and disks following XRF interrogation

The reason for this significant difference in dissolution compared to iridium powder, may be because iridium targets are often either platinum coated to attenuate beta activity, or are alloyed with platinum to generate softer metals. However, analyses of the target surfaces using XRF spectroscopy eliminated this possibility, because apart from the *K*_*α*1/2_ peak of the instruments molybdenum anode at 17.41 keV, the only peaks present in the spectra are the characteristic *L* and *M* lines of iridium (Fig. 4). It was therefore suggested that this drop in recovery is a direct consequence of the differences in sample oxidation that are caused by the much smaller surface area to volume ratios of the two target materials.

To counteract this, trials where the targets are crushed using a boron carbide pestle and mortar prior to digestion have been performed. These resulted in a marked rise in dissolution (Table 4). Although a pestle and mortar is not the most efficient technique, these trials do suggest that provided the target material structure can be sufficiently degraded prior to digestion, full target dissolution is possible.

**Table 4:** Difference in iridium recoveries caused by crushing target materials prior to dissolution

Crushing	Iridium dissolved (%)	
	Pellet	Disc
Pre	1.40 ± 0.65	3.85 ± 1.80
Post	6.10 ± 0.40	14.1 ± 2.40

### Target material impurities

To ensure the smallest source size with the largest possible specific activity, the chemical purity of iridium targets must be high; most manufacturers state their material is 99.9% pure. The impurities that are present within target materials prior to irradiation though have a direct impact on computational studies that model source production, because they could potentially mask or skew the signatures of interest. Quantifying these nuclides, and incorporating them into our models, would assist in further refining which impurity nuclides could be used as nuclear forensic indicators.

To this end, ICP-MS has been used to make initial impurity assessments of the QSA Global targets (Table 5). This was made possible following the total digestion of the target materials using a series of dissolution steps in combination with the improvements stated above. In general, the impurity levels in the pellets and disks are low. However, the impact that significant quantities of platinum and tungsten (key iridium neutron transmutation products) have on signature development needs to be investigated further. As does the possibility of copper being used as an independent irradiation indicator; if the levels seen here can be proven to be consistent between targets generated by different manufacturers.

**Table 5:** Concentration of selected elemental impurities within QSA Global iridium target materials

Target	Elemental concentration (μg g^−1^)									
	Ag	Au	Cu	Os^a^	Pd	Pt^b^	Re	Rh	Ru	W
Pellet	0.80 ± 0.05	0.20 ± 0.05	19.0 ± 3.00	1.30	1.20 ± 0.10	34.0 ± 3.00	0.70 ± 0.05	10.0 ± 0.25	5.90 ± 0.15	6.10 ± 0.50
Disk	4.60 ± 0.10	0.10 ± 0.01	55.0 ± 8.00	2.80	0.90 ± 0.05	15.0 ± 1.00	1.70 ± 0.05	1.70 ± 0.05	0.60 ± 0.05	12.0 ± 0.50

^a^Osmium measurements are only semi-quantitative, because of the volatile nature of the osmium oxide formed during the dissolution process

^b^Due to iridium hydride interference, platinum measurements were made using only the ^196^Pt and ^198^Pt isotopes

## Conclusion

Total dissolution of iridium powder can be achieved when H_2_O_2_ is used as an oxidising agent during pressure digestion in c.HCl, and results in the production of hexachloroiridate(IV). Instances of incomplete sample digestion result in the production of hexachloroiridate(III) in addition to hexachloroiridate(IV), but can be counteracted through the addition of further H_2_O_2_ post digestion. Preliminary tests on iridium source materials showed minimal dissolution, however modifying the reaction conditions and crushing the targets prior to digestion gave rise to a marked increase in dissolution. Provided the structure of the target material can be sufficiently degraded prior to digestion, these results suggest that full target dissolution is possible. This was substantiated by impurity evaluations that were performed on QSA Global radiography source targets following digestion using a series of dissolution steps.
